# Combining capture–recapture data and known ages allows estimation of age‐dependent survival rates

**DOI:** 10.1002/ece3.4633

**Published:** 2018-12-27

**Authors:** Tomas Bird, Jarod Lyon, Simon Wotherspoon, Charles Todd, Zeb Tonkin, Michael McCarthy

**Affiliations:** ^1^ Department of Botany Center for Excellence in Environmental Decisions University of Melbourne Melbourne Vic. Australia; ^2^ Department of Fisheries and Oceans NorthWest Atlantic Fisheries Centre St John's Newfoundland Canada; ^3^ Department of Sustainability and Environment Arthur Rylah Institute Melbourne Vic. Australia; ^4^ Institute of Marine and Antarctic Studies University of Tasmania Hobart Tas. Australia

**Keywords:** age, Bayesian, individual growth, otoliths, state‐space, survival

## Abstract

In many animal populations, demographic parameters such as survival and recruitment vary markedly with age, as do parameters related to sampling, such as capture probability. Failing to account for such variation can result in biased estimates of population‐level rates. However, estimating age‐dependent survival rates can be challenging because ages of individuals are rarely known unless tagging is done at birth. For many species, it is possible to infer age based on size. In capture–recapture studies of such species, it is possible to use a growth model to infer the age at first capture of individuals. We show how to build estimates of age‐dependent survival into a capture–mark–recapture model based on data obtained in a capture–recapture study. We first show how estimates of age based on length increments closely match those based on definitive aging methods. In simulated analyses, we show that both individual ages and age‐dependent survival rates estimated from simulated data closely match true values. With our approach, we are able to estimate the age‐specific apparent survival rates of Murray and trout cod in the Murray River, Australia. Our model structure provides a flexible framework within which to investigate various aspects of how survival varies with age and will have extensions within a wide range of ecological studies of animals where age can be estimated based on size.

## 1 INTRODUCTION

Characterizing individual variability in population dynamic rates is an important consideration in ecological studies. For example, age‐dependent survival is an integral aspect of population dynamics (Chaozhi, Ovaskainen, Saastamoinen, & Hanski, 2007; McCrea, Morgan, & Cole, 2013). In many species, survival rates can vary by orders of magnitude across all age classes, or can change markedly at various life‐history stages. This correlation between age and survival is likely linked to size, as older individuals tend to be larger and less vulnerable to predation. On the other hand, all animal species have a life span that is limited by aging rather than extrinsic factors (Péron et al., 2010). Understanding this variation in survivorship across ages can provide information on population dynamics such as the proportion of new recruits that will survive to reproductive age, impacts of management interventions on population dynamics, and how to target harvesting efforts to maximize sustainability (Berkeley, Hixon, Larson, & Love, 2004).

Our motivation for wanting to understand size and age‐related survival came from a study focussed on the population dynamics of trout cod (*Maccullochella macquariensis* Mitchell) and Murray cod (*Maccullochella peeli* Mitchell) in the Murray River, Australia. Populations of both Murray cod and trout cod are thought to be at <10% of pre‐European settlement levels (at the time of this study), and the trout cod population is primarily limited to a 95 km stretch of river in Victoria, Australia (Allen, Brown, Douglas, Fulton, & Catalano, 2009). While trout cod are a protected species, Murray cod continue to be fished recreationally, with anglers allowed to take individuals in the slot size of 600 and 1,000 mm (Allen et al., 2009). However, as trout cod are similar in appearance to Murray cod and hunt similar prey items (Ebner, 2006), they are at risk of being caught and damaged or killed by mistake. In both species, fish within the legal size window clearly have an elevated risk of mortality. Murray cod, which grow more quickly through this size window than trout cod (Anderson, Morison, & Ray, 1992; Koehn & Harrington, 2006), will therefore have a reduced overall risk of death. In managing the competing interests of the active recreational fishery and the conservation of a species, the relative size and ages of the populations of these two species will be an important consideration.

There are several commonly used approaches estimating of age‐specific survival for populations in the wild. Capture–mark–recapture (CMR) studies are frequently used when juveniles can be identified and tagged before release (Bouwhuis, Choquet, Sheldon, & Verhulst, 2012; Chilvers & Mackenzie, 2010; Meixell et al., 2013; Schmaltz, Cezilly, & Bechet, 2011). For species that can be aged using internal structures, sacrificing a subset of the population in a CMR analysis can also provide a solution (Francis & Campana, 2004). Finally, a number of approaches for developing size and age‐related survival have been developed in the fisheries stock‐assessment scenarios in which catch and effort data are available (Fournier, Hampton, & Sibert, 1998; Fournier, Sibert, & Terceiro, 1991; Maunder & Punt, 2013). Multistate CMR models have been used to infer transitions between clearly defined developmental or life‐history stages (Pollock, 1981), and Leslie matrix models have been used in such cases (Buckland, Newman, Thomas, & Koesters, 2004). Size has sometimes been used as a means of marking stages in a life history, as fecundity or vulnerability to predation is both related to size (Begg, Hare, & Sheehan, 1999).

Recent advances in estimating age‐dependent survival rates include a mixture modeling approach which requires that ages of some of the population are known (McCrea et al., 2013). Alternate approaches assume that the shape of the survival‐at‐age curve follows a known distribution such as the Weibull to infer the survival rates at different ages (Colchero, Jones, & Rebke, 2015; Matechou et al., 2013). While reasonable in many situations, true survival parameters in wild populations may be subject to variation that is not readily explained by such models. Furthermore, it may be that the variation in survival rates is better described by a mixture of distributions or nonlinear functions.

Where a species’ growth follows a well‐defined curve, it may be possible to fit a growth model without knowing the ages of individuals a priori by observing the change in size of individuals between successive capture occasions. Past work has shown how modeling growth in a CMR framework can be accomplished by treating observed changes in size as realizations of random normal processes (Bonner & Schwarz, 2006). As well, Eaton and Link (2012) developed models allowing the estimation of age at first capture based on a number of growth curves.

We show how assuming that individual growth follows a well‐defined trajectory, such as the von Bertalanffy (VBF) growth function (Von Bertalanffy, 1957), allows us to estimate age at first capture based on estimated growth parameters, meaning that all subsequent ages are known. By incorporating this model for age at first capture into a multistate Cormack–Jolly–Seber (CJS) model, it is possible to estimate survival at age for the remainder of the study. We first outline the model structure for both the growth and survival models and show how the growth model is able to accurately estimate the ages of individual fish using CMR, as compared to ages of fish that derived by counting rings on their otoliths. We then show how it is possible to recover age‐dependent survival and capture rates using both the Murray River and simulated datasets.

## 2 METHODS

### 2.1 Data collection

Murray cod and trout cod were sampled during a large‐scale sampling project over a period of 6 years in the Murray River, Australia. Sampling was undertaken using 5 and 7.5 KW generator powered pulsator (GPP) Smith‐Root boat‐mounted electrofishing units. Between 100 and 123 sites with average size of 10,000 m^2^ were sampled annually from 2007 to 2012 between April and June each year. All fish captured were weighed (to the nearest gram) and measured (total length [TL] to the nearest millimeter). A uniquely coded external t‐bar or dart tag was inserted on the left side of each fish (over 200 mm TL), adjacent to the dorsal fin. Additionally, each fish (over 250 mm TL) was also implanted with a uniquely numbered passive integrated transponder (PIT) tag in the shoulder below the dorsal fin.

### 2.2 Model development

Our model development sits in the general field of state‐space models, in which the observation data (captures and lengths of individual fish) are dependent on partially observed processes of survival and growth. Our general approach is to adopt a standard CMR framework, with the addition of a growth model which provides estimation of a latent age variable. We first describe down the growth model followed by the CMR model.

#### 2.2.1 Growth model

We use the VBF growth function to describe a length‐at‐age curve for Murray cod. We assume that the age structure of the population is well represented by sampling, though we account for the fact that capture probabilities for each age may vary. We first estimate growth parameters based on the observed changes in length for all individuals that are ever recaptured once or more. Next, we estimate the age at first capture for every individual in the population and include these as a covariate in a state‐space CJS model.

We start with *k* = 6 evenly spaced capture occasions at times t=1,⋯,k and the *M* fish that are ever captured during sampling are labeled i=1,⋯,M. The times of first capture for each fish are denoted *f*_*i*_ (i=1,⋯,M). The last capture occasion for each fish is denoted $q_{i}$ (i=1,⋯,M), and $C_{i}$ is the number of times each individual was captured. For each *i*, we observe a series of captures $y_{i}=\left(y_{if_{i}},\;\cdots ,\;y_{\mathrm{ik}}\right)$ where $y_{\mathrm{it}}=1$ if fish *i* was captured and 0 if not. In addition, we record a vector $a_{i}=\left(a_{if_{i}},\;\cdots ,\;a_{\mathrm{ik}}\right)$ indicating whether or not fish *i* was alive on each of the capture occasion and where $a_{\mathrm{it}}=1$ if a fish was observed alive or can be assumed to be alive based on capture in a later sample.

To model growth, we also record the measured lengths $L_{\mathrm{it}}$ ($t=f_{i},\;\cdots ,\;q_{i}$), on each occasion individual *i* was captured. As only individuals that are recaptured following $f_{i}$ provide data on growth rates, we denote the set G as those individuals that were ever recaptured following $f_{i}$. We record $w_{1}\;\cdots ,\;w_{C_{i}}$ (i∈G) as the interval between each recapture and $g_{\mathrm{ic}}$ $\left(c=1,\;\cdots ,\;C_{i}-1;i\in G\right)$ is the observed change in size. We model growth based on a two‐parameter VBF function for asymptotic growth introduced by Beverton (1954) and Beverton and Holt (1957), where the length of individual i at time *t* is given by

(1) $$L_{\mathrm{it}}=L_{\infty }-\left(L_{\infty }-L_{0}\right)\left(1-e^{-Ks_{i}}\right),$$

and $L_{\infty }$ is the asymptotic size, $L_{0}$ is defined here as 100 mm here based on the size of Murray cod and trout cod young‐of‐year (Koehn & Harrington, 2006), *K* is the growth parameter, and $s_{i}$ is the age of individual *i*. In CMR data, lengths are only observed for captured individuals, but we can estimate the parameters *K* and $L_{\infty }$ based on changes in the lengths of individuals. Rearranging (1) the observed changes in size between *t*_1_ and *t*_2_ is

(2) $$g_{\mathrm{ic}}=\left(L_{\infty }-L_{i1}\right)\left(1-e^{-K(\omega_{\mathrm{ic}})}\right).$$

If we assume that each value in $g_{i}$ is an exchangeable, normally distributed random variable, we can use 2 to estimate growth parameters *K* and $L_{\infty }$. The joint posterior for the parameters given the data is

$$\left[K,L_{\infty },\sigma |g_{i},w_{i}\right]\propto \sum_{c=2}^{C_{i}}\left[g_{\mathrm{ic}}|K,L_{\infty },\sigma ,w_{i}\right]\left[K,L_{\infty },\sigma \right]$$

and the process giving rise to the observed change in lengths can be written as

$$g_{\mathrm{ic}}|K,L_{\infty },\sigma ,w_{i}∼\text{Normal}\left(\left(L_{\infty }-L_{ic-1}\right)\left(1-e^{-K(w_{\mathrm{ic}})}\right),\sigma^{2}\right)$$

where $\sigma^{2}$ is the variance of the normal distribution. We provide a common Uniform(0, 10,000) prior for $L_{\infty }$. For the growth parameter *K*, we recognize that some fish have different growth trajectories and thus allow each individual to have a separate growth parameter $K_{i}$, which we assume to be drawn from a Beta distribution with

$$K_{i}∼\text{Beta}\left(\alpha ,\beta \right)$$

where α and β both have Gamma(0.01,0.01) hyperpriors.

Given *K*_*i*_ and $L_{\infty }$, we can then derive an age at first capture $s_{if_{i}}$ for each individual in the study, similar to the approach of Eaton and Link (2012).

(3) $$s_{if_{i}}=-\frac{\text{log}\left(\frac{L_{\infty }-L_{0}}{L_{\infty }-L_{if_{i}}}\right)}{K_{i}},$$

and the ages for the rest of the study follow from $s_{if_{i}}$. In this context, we generate a distribution for each $s_{i}$, which we keep as a continuous variable and use the distribution for each $s_{if_{i}}$ as prior information for the age at first capture in the CMR component.

#### 2.2.2 Capture and survival conditional on age

To fit the assumptions of a CMR framework, we start with setting sampling intervals at regular yearly occasions over the 6‐year period. We assume no temporary migration in the population between years and that permanent emigration is confounded with survival. The joint posterior distribution for the individual capture ($p_{\mathrm{it}}$) and survival ($\phi_{\mathrm{it}}$) parameters given the data is

$$\left[p,\phi |y,a,s\right]\propto \prod_{i=1}^{N}\left[a_{if_{i}}\right]\prod_{t=f_{i}+1}^{T}\left[y_{\mathrm{it}}|a_{\mathrm{it}},p_{\mathrm{it}}\right]\left[a_{\mathrm{it}}|a_{it-1},p_{\mathrm{it}},\phi ,s_{\mathrm{it}}\right]\left[p_{\mathrm{it}}\right]\left[\phi \right]\left[s_{\mathrm{it}}\right],$$

where $s_{\mathrm{it}}$ is the age at first capture derived from Equation [Link]. Assuming that the observed captures are Bernoulli distributed random variables,

$$y_{\mathrm{it}}|a_{\mathrm{it}},p_{\mathrm{it}}∼\text{Bernoulli}\left(\tau_{\mathrm{it}}\right)$$

where $\tau_{\mathrm{it}}=a_{\mathrm{it}}p_{s_{\mathrm{it}}}$ and $p_{s_{\mathrm{it}}}$ is the age‐specific probability of capture. We can incorporate age in the probability of capture using either separate values of $p_{s_{\mathrm{it}}}$ for each age or with a logistic regression to model a linear relationship between age and capture. In order to minimize the number of parameters estimated in the model, we choose the latter approach with

$$\text{logit}\left(p_{\mathrm{it}}\right)=\beta_{0}+\beta_{1}s_{\mathrm{it}}$$

and the logit function is

$$\text{logit}\left(p\right)=\text{log}\left(\frac{p_{\mathrm{it}}}{1-p_{\mathrm{it}}}\right).$$

We next assume that the elements of $a_{i}$ are exchangeable Bernoulli distributed random variables so that the process of surviving from time t-1 to time *t* is described by

$$a_{\mathrm{it}}|a_{it-1},\phi_{s_{it-1}}∼\text{Bernoulli}\left(a_{it-1}\phi_{s_{it-1}}\right)$$

where $\phi_{s_{it-1}}$ is the probability of surviving from age t-1 to age *t*. We first used a simple logit‐linear model for how survival (or detection) varies to model the expectation that survival generally increases as fish grow older,

$$\text{logit}\left(\phi_{s_{\mathrm{it}}}\right)=\rho_{0}+\rho_{1}s_{\mathrm{it}}.$$

We use Normal (0, 0.0001) priors for parameters $\beta_{0}$, $\beta_{1}$, $\rho_{0}$, and $\rho_{1}$.

We were also interested in the impact of fishing on age‐dependent survival rates, and whether we could discern a change in survival associated with the size window within which anglers are allowed to keep trout cod. As anglers in the Murray River are allowed to keep Murray cod between 600 and 1,000 mm (though these limits have varied somewhat over time), fish within this window may be subject to greater recreational angling pressure. A simple linear relationship may be inadequate to describe variation in survival with age. We tested quadratic and third‐order polynomial functions to examine how survival varied with age. In addition, we tested simple linear and quadratic functions for how capture probabilities varied with age.

### 2.3 Incorporating known‐age data

We were able to compare our estimates of individual age against a reference dataset of individuals with known age from the same populations as those used in the CMR study. A sample of fish of representative sizes was collected each year as a part of a larger monitoring program. All fish collected were euthanized and measured for TL (nearest mm), and their sagittal otoliths were removed, dried, and stored before embedding in epoxy resin. Otoliths were then sectioned through the primordium across the transverse plane, mounted on a glass slide with thermoplastic cement, and polished using 3‐μm lapping film to remove major scratches. Sections were independently labeled and photographed at 25× magnification using a dissecting microscope illuminated with transmitted light. Ages were estimated by multiple readers by counting the completed zones (translucent–opaque sequence) without information on fish size or sampling date to estimate an individual's age in years (Mallen‐Cooper & Stuart, 2003). Where readers disagreed, a third reading was conducted.

We first used these known‐age fish to independently estimate growth parameters based on Equation (1), assuming that the sacrificed fish represented a random subset of the trout cod and Murray cod populations. Second, we compared these growth parameters based on known‐age fish to those estimated from observed length changes in the CMR‐only model. Finally, we developed a hybrid model that used both known ages and the observed length changes to estimate a third set of growth parameters.

### 2.4 Simulations

We conducted a series of simulations to determine how well we could estimate parameters describing how survival and detection probabilities vary with age, as well as the true ages of individuals. Our simulation scenario used the growth equations described in Equation (1) to generate growth trajectories for a population of 1,000 fish with an initial size distribution described by a negative binomial distribution. We assumed a linear relationship for both survival and detection with age, and then simulated capture–recapture sampling over 10 evenly spaced occasions. We then used the CMR models described above to try and recover survival, capture, and growth parameters from the data. To test the robustness of the model to variation in data quality across ages, we first estimated survival and detection parameters assuming no dependence between ages—that is the survival and detection parameters were estimated independently. We then repeated the simulation but allowed a linear model as in Equation (4).

### 2.5 Bayesian inference

We used Markov chain Monte Carlo (MCMC) sampling software JAGS via the R package “R2jags” (Plummer, 2003; R Development Core Team, 2013; Su & Yajima, 2011) to generate samples from the joint posterior distribution for each model, running three chains of 150,000 iterations each, with a burn‐in period of 100,000, keeping every 50th iteration for a total of 3,000 iterations. Convergence was assessed both visually and using the Gelman–Rubin diagnostic.

## 3 RESULTS

### 3.1 Data summary

Summaries of CMR data for the 9 years of surveys are presented in Tables 1 and 2. Between 105 and 154 surveys were conducted each year between 2005 and 2013, yielding captures of between 252 and 1,388 trout cod and 106 to 899 Murray cod. Captured trout cod were on average around 300 m in size, ranging from a mean of 260 ± 73 mm in 2005 to 375 ± 77 mm in 2013 (Table 1). Murray cod were generally larger, with an average length of 427 ± 77 mm and mean length in each year ranging from 335 ± 153 mm to 568 ± 194 mm (Table 2).

**Table 1:** Summary statistics for sizes, numbers, and recaptures of trout cod from electrofishing captures. Intervals are presented in standard deviations

Year	Surveys	Captured	Recaptures	Mean length (mm)
2005	105	1,388	0	260 ± 73
2006	121	1,264	75	268 ± 72
2007	130	730	102	293 ± 73
2008	148	887	108	285 ± 75
2009	151	881	114	285 ± 75
2010	145	785	107	299 ± 65
2011	114	252	46	353 ± 78
2012	154	916	134	353 ± 82
2013	139	368	102	375 ± 77

**Table 2:** Summary statistics for sizes, numbers, and recaptures of Murray cod from electrofishing captures. Intervals are presented in standard deviations

Year	Surveys	Captured	Recaptures	Mean size (mm)
2005	107	899	0	335 ± 153
2006	121	820	138	348 ± 155
2007	130	514	168	373 ± 160
2008	144	472	207	403 ± 179
2009	142	325	238	398 ± 173
2010	124	244	163	431 ± 162
2011	92	106	60	453 ± 195
2012	127	222	97	568 ± 194
2013	123	242	90	426 ± 220

Summaries of the sizes and ages of sacrificed trout cod and Murray cod are presented in Tables 3 and 4. Length‐frequency histograms of the sizes of captured trout cod and Murray cod are presented in the supplementary materials. The average sizes of sacrificed fish were similar to those of captured fish.

**Table 3:** Summary statistics for sacrificed trout cod

	Mean length	# otoliths	Mean age
2007	240 ± 123	41	4 ± 3.9
2008	206 ± 48	32	2.3 ± 1.3
2009	209 ± 76	18	2.6 ± 2.2
2010	158 ± 70	7	1.3 ± 1.8
2011	325 ± 181	5	5.2 ± 4.8
2012	360 ± 107	20	7.9 ± 4.6

**Table 4:** Summary statistics for sacrificed Murray cod

	Mean length	# otoliths	Mean age
2008	412 ± 155	3	4.7 ± 3.1
2009	446 ± 54	5	6.4 ± 1.1
2010	574 ± 28	2	6.5 ± 0.7
2011	329 ± 306	8	3.6 ± 4.6
2012	582 ± 132	5	9.4 ± 3.4

### 3.2 Estimated versus true ages of otolith‐aged fish

**Figure 1:**
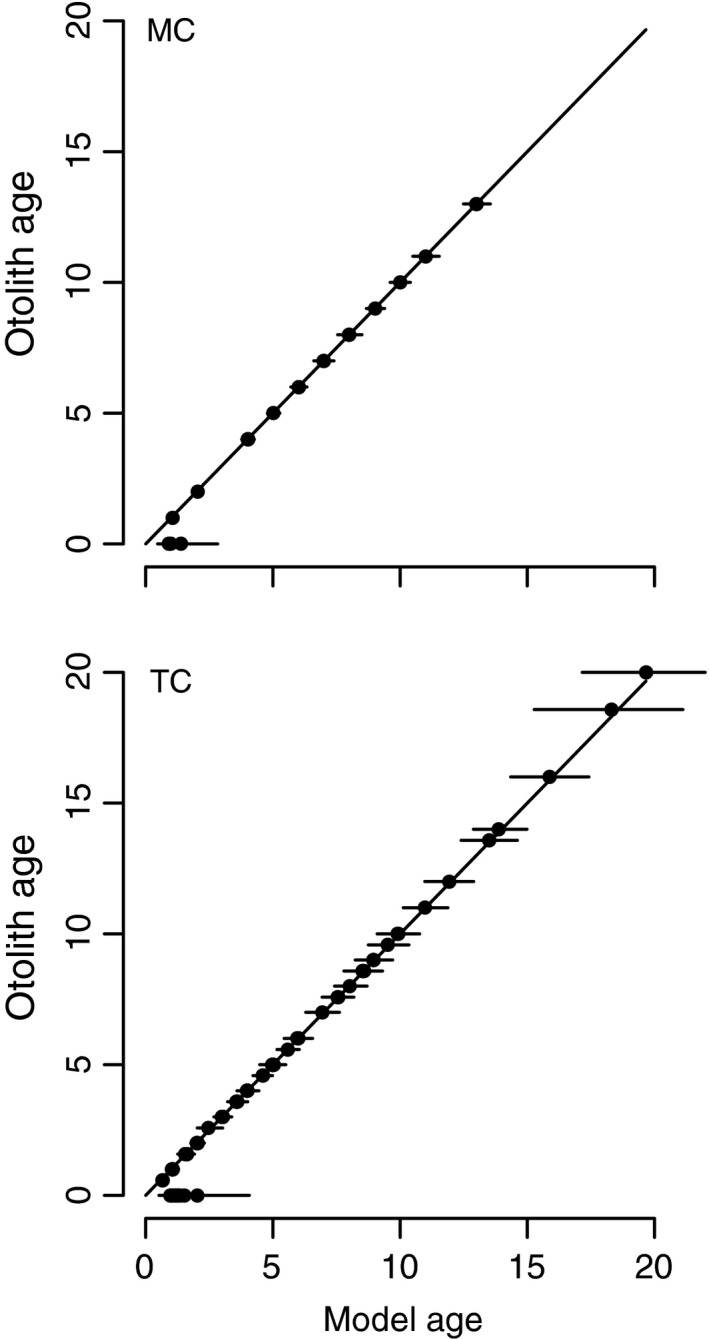
Comparison of ages for Murray cod (MC) and trout cod (TC) measured from otoliths versus ages back‐calculated using Equation (3) and the growth parameters estimated from the joint otolith—CMR model. Solid line is the 1:1 line, and segments indicate 95% credible intervals around estimated ages

**Figure 2:**
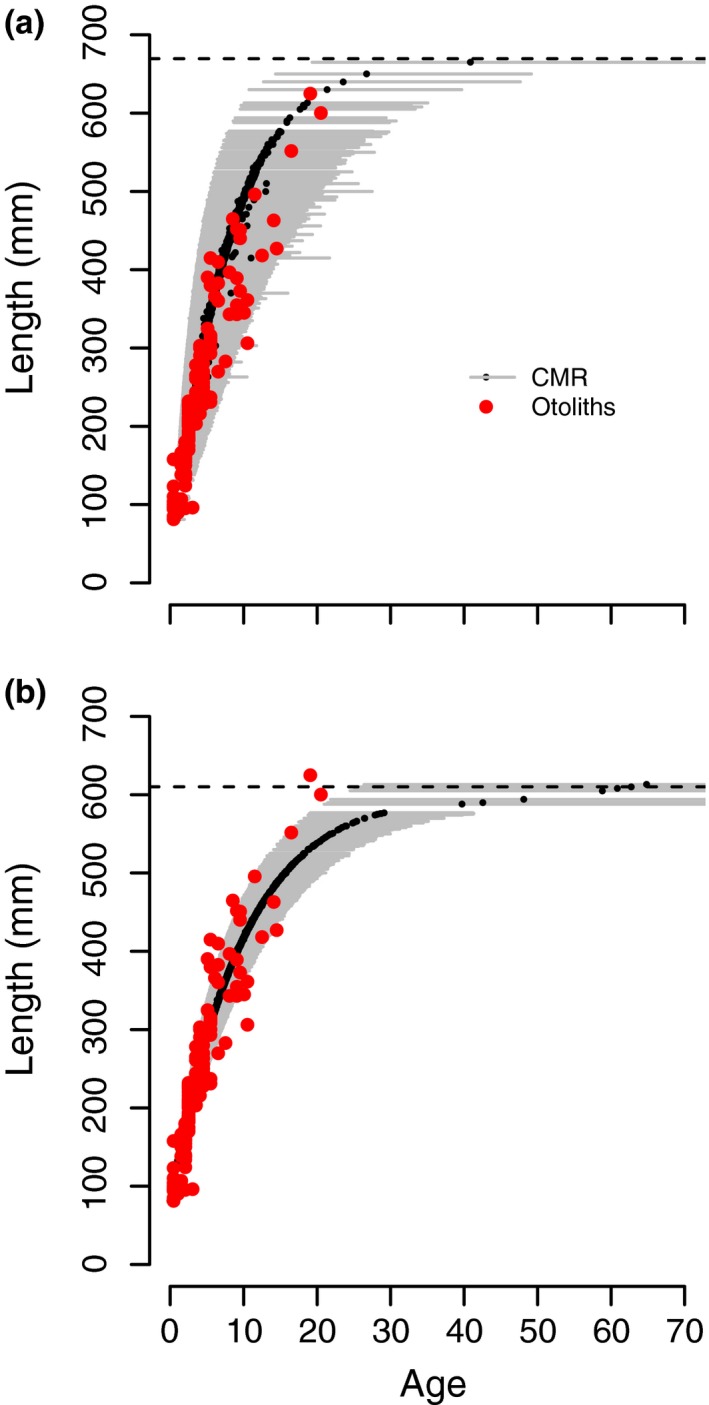
Estimated ages at first capture for trout cod in Capture–mark–recapture (CMR) data based on (a) a joint analysis of CMR and otolith data and (b) CMR data alone. Each black point represents the mean of the posterior distribution for each individual's age, while horizontal segments indicate 95% credible intervals around that age. Red dots indicate the ages of individual fish estimated from otoliths, and the horizontal dashed line represents *Ł*_∞_

**Figure 3:**
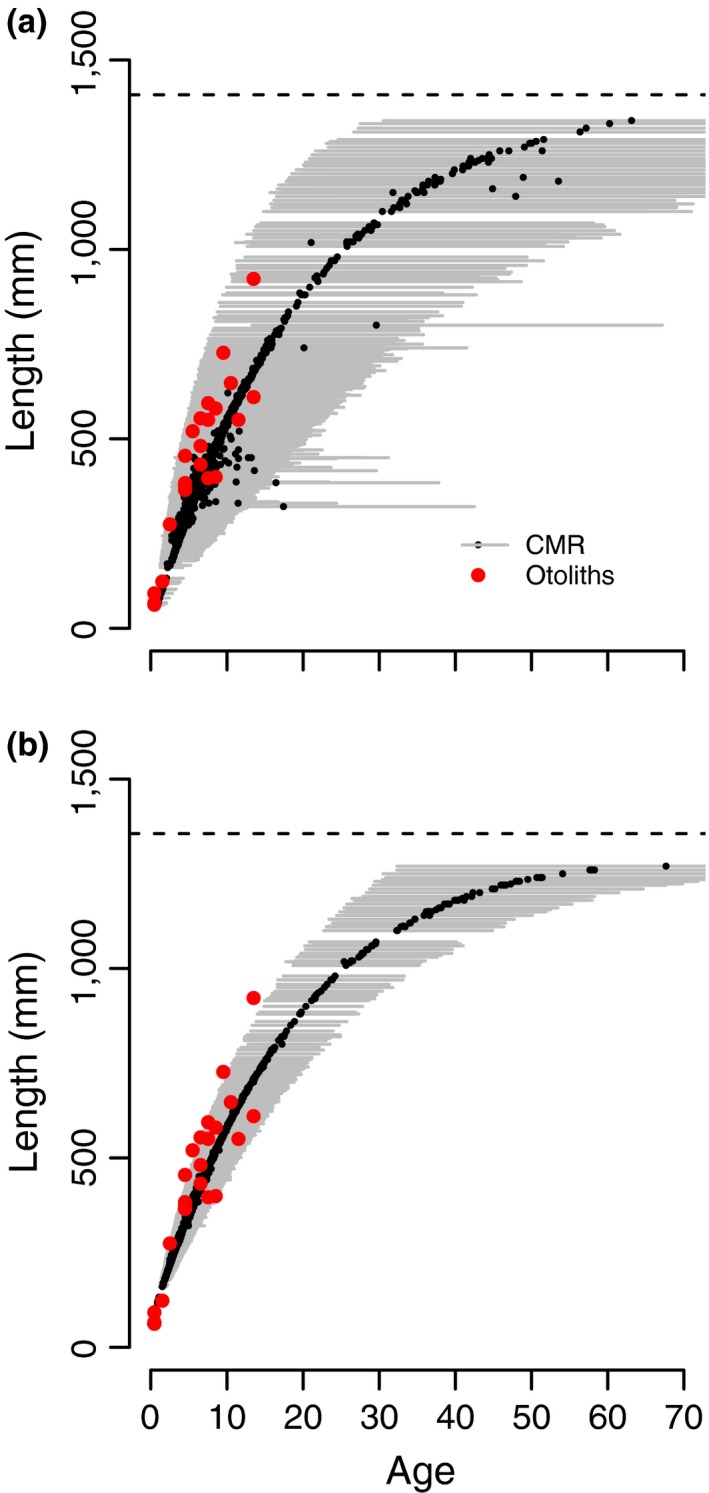
Estimated ages at first capture for Murray cod in CMR data based on (a) a joint analysis of CMR and otolith data and (b) CMR data alone. See Figure 2 for a description of symbols

Using an individually varying random parameter for the growth rate *K* meant that there was no discrepancy between estimated ages and true ages, except for the youngest and oldest fish (Figure 1). The inclusion of these known ages in the age‐specific survival model had a strong influence on estimated age structures for both fish. For both trout cod (Figure 2) and Murray cod (Figure 3), the model based on CMR data alone had 95% Bayesian credible intervals that in some cases did not overlap with known ages. By contrast, the CMR model had broader credible intervals that included almost all known ages.

**Figure 4:**
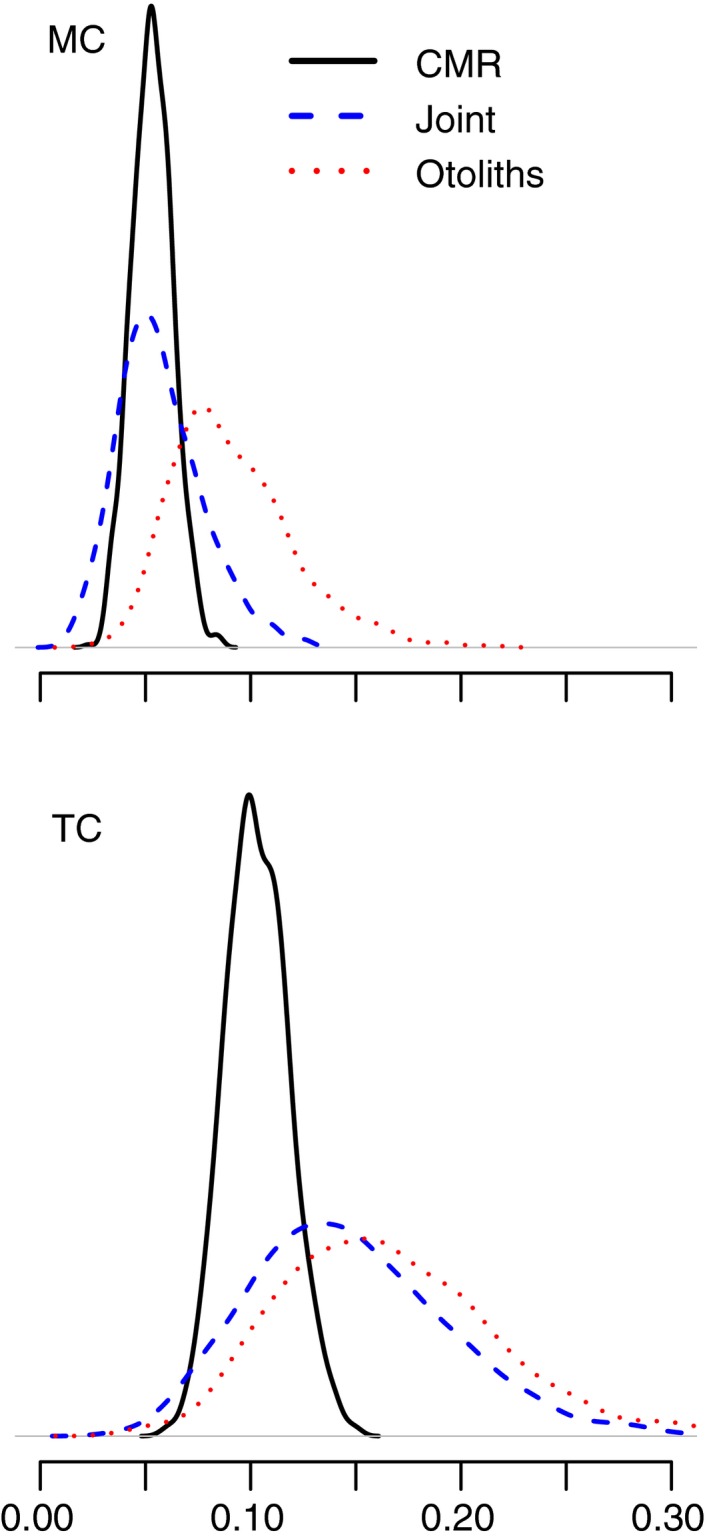
Density for the posterior distribution of the growth parameters $K_{i}$, as calculated from otolith data alone (red), CMR data alone (black), and a joint analysis of both (blue)

The inclusion of otolith data generally increased the mean of the distribution of the Beta‐distributed $K_{i}^{\prime }s$ for both species, though this effect was stronger in trout cod, where there were more otoliths used in the analysis (Figure 4). This effect can be seen in the length‐at‐age curves, which are generally steeper in the joint model.

### 3.3 Age‐dependent survival and detection

**Figure 5:**
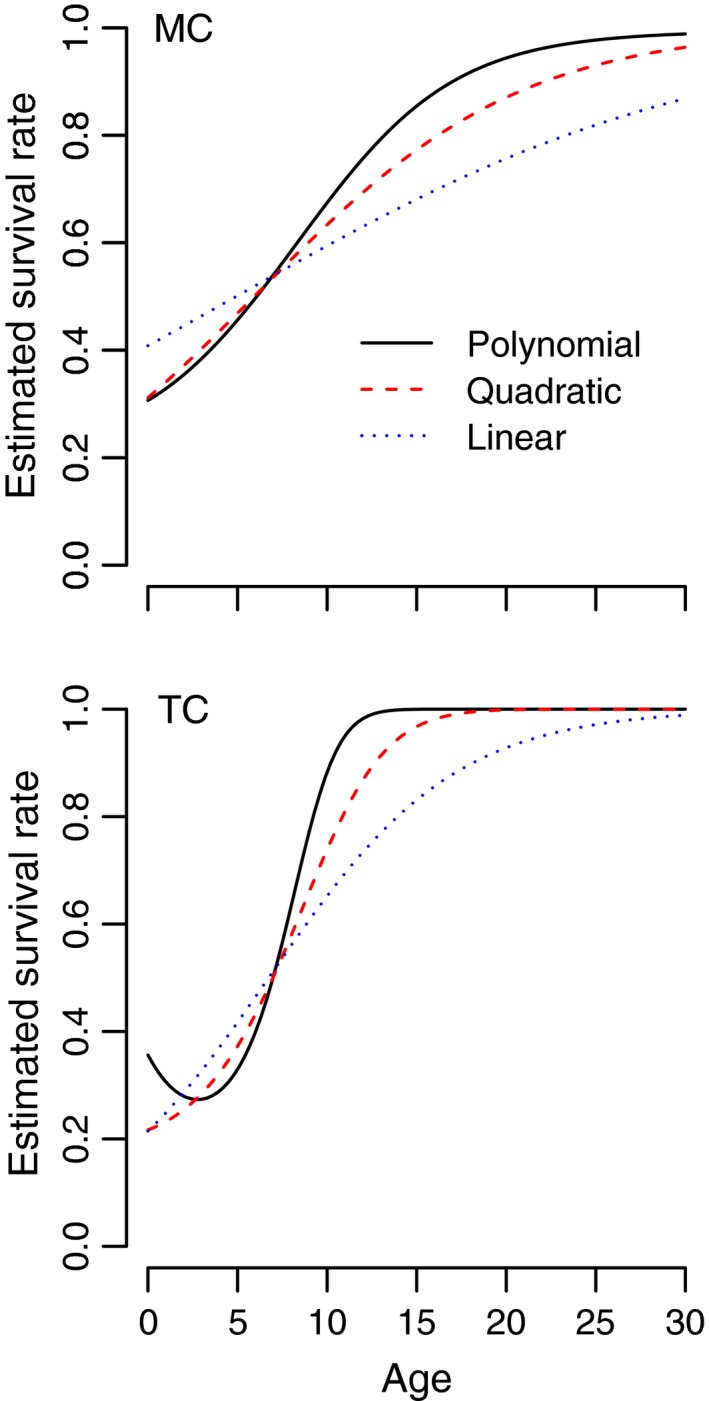
Estimates of age‐dependent survival rates for trout cod (TC) and Murray cod (MC) based on linear, quadratic, and polynomial models

We find that for both Murray cod and trout cod, survival generally increased as a function of age, though for trout cod there was weak support for a polynomial relationship between age and survival (Figure 5). Similarly, detection probabilities for both species were generally increasing. DIC values for each model revealed that linear models for survival and detection were ranked far higher than any quadratic or polynomial forms (by at least 100 DIC points in all cases).

### 3.4 Simulations

**Figure 6:**
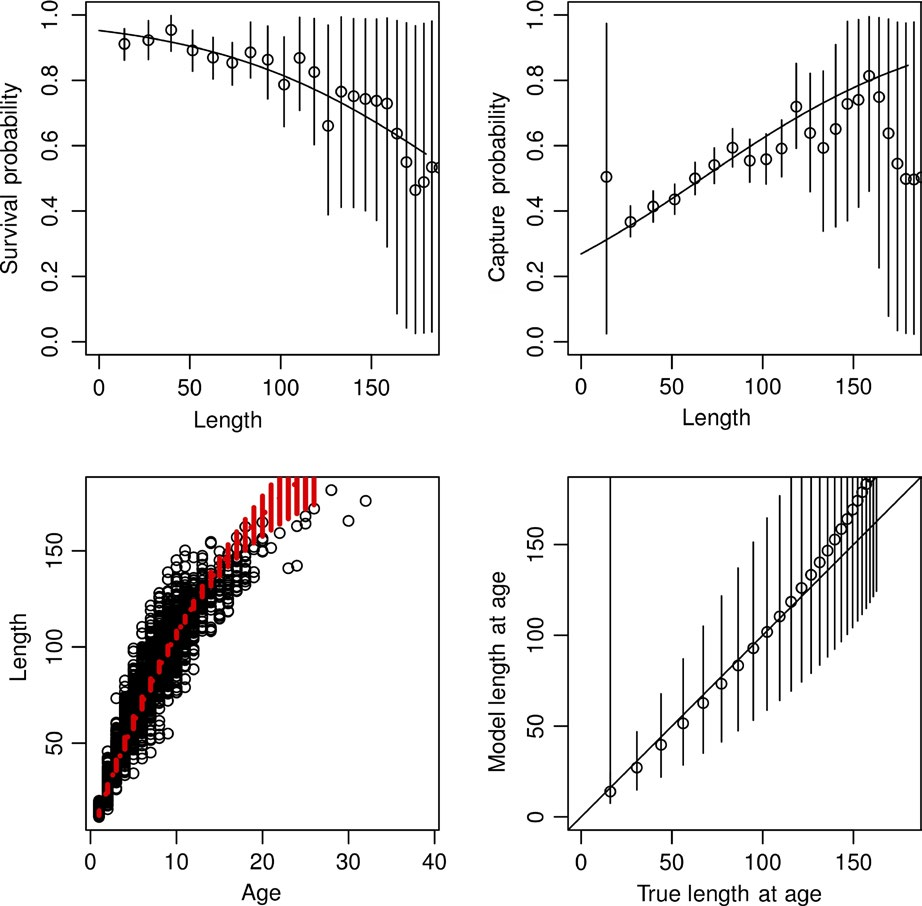
Relationship between parameters estimated from an age‐specific capture model and true values used to generate simulated data. In the simulated data, both survival and capture probabilities are linearly dependent on age, but in the model, age‐specific rates are given uninformative Uniform(0,1) priors for age‐specific survival and capture probabilities. For survival, detection, and model versus true length plots, each point represents the model estimated value at for each age, while vertical lines indicate 95% credible intervals around each estimate. For length versus age, points represent the simulated data, while the red line indicates the estimated relationship between length and age, based on the von Bertalanffy growth equation

**Figure 7:**
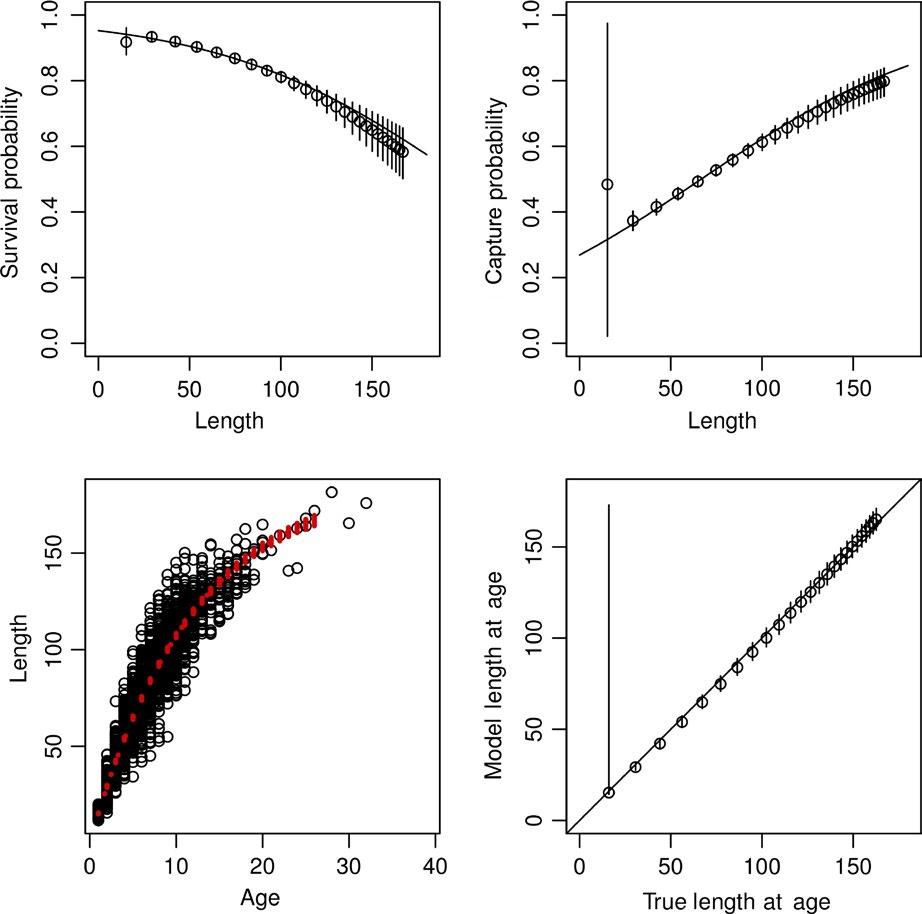
Estimated parameters and simulated data using an age‐dependent survival and capture probability model in which both survival and capture probability are linearly dependent on age. See Figure 6 for an explanation of symbols

Simulations show that ϕ estimated independently for each age are close to the true values only for those ages that had sufficient data (Figure 6a,b), and estimated survival rates for ages with small sample sizes were poorly estimated with large credible intervals. Similarly, estimates of size at age and population‐level growth rates are close to the true values (Figure 6c,d), but vary increasingly with larger sizes. When we used a parametric model describing how survival varied with the average size of individuals in each age class, the model results were very close to the true values (Figure 7). As well, we found that estimated ages were much more accurate using the parametric model.

## 4 DISCUSSION

Our analyses demonstrate the feasibility of using growth models within a capture–recapture framework to estimate age‐specific survival and recapture rates. Doing so makes use of the information available in sequential observations of animal size without requiring assumptions about the underlying shape of the survival curve, allowing for flexible specification of models that account for age‐specific factors such as phenology, migrations, density dependence, or otherwise. At the same time, our age estimates acknowledge the variability and uncertainty in growth rates, which then carries through to uncertainty in our estimated survival and detection rates. By specifying models for capture rates, we are also able to attribute individual variability in detection to age‐specific factors, which may help eliminate an important source of bias where different‐aged animals are greatly different in their recapture rates. By using a state‐space framework (King, 2012), the method is easily applied to other applications.

For Murray cod, the growth rates $K_{\mathrm{mean}}=0.055$ and $L_{\infty }=1,375\;\text{mm}$ estimated by our joint CMR–otolith and CMR‐only models compared well previously published estimates; Rowland (1998) found K=0.06 and $L_{\infty }=1,369\;\text{mm}$ in a variety of populations located in NSW and Victoria. For trout cod, our estimated $L_{\infty }=608\;\text{mm}$ compared favorably with the $L_{\mathrm{infty}}=582\;\text{mm}$ estimated by Lyon et al. (2012). However, our estimated $K_{\mathrm{mean}}=0.10$ for CMR data and $K_{\mathrm{mean}}=0.14$ for otolith data were significantly lower than the $K_{\mathrm{mean}}=0.19$ found in their study. We emphasize in this case that different populations have been observed to have widely varying growth rates, so some difference in these parameters is to be expected. We also note that for Murray cod, the asymptote $L_{\infty }$ was markedly higher than the largest observed fish. This potentially reflects the paucity of data from larger fish. A further minor difference between models for trout cod and Murray cod lays in how the inclusion of otoliths influenced the width of the credible intervals around estimated ages. For trout cod, credible intervals were narrower in the combined model than for the combined model for Murray cod. This was likely due to the fact that the sample size of otoliths for Murray cod was much smaller.

One of the key advantages of our approach is that we allow survival and capture rates to be flexibly modeled as a function of age. In the Murray cod data, we explored simple polynomial functions, and in the case of both Murray cod and trout cod, we find that age is significantly related to both survival and growth. The top‐performing models for Murray cod turned out to be simple linear relationships with age. This is likely due to the fact that the greatest sources of mortality are in the first years of age, both from natural and fishing mortality. In addition, variability in the length‐at‐age relationship means that the distinct legal boundary of 600 mm for Murray cod corresponds to ages between 5 and 15 years of age. Prior to this age, natural mortality is likely high and may incorporate some level of mortality due to the stresses of being captured and released. When Murray cod have grown beyond the age at which they reach a maximum legal size (at 21 [10, 50] years), survival is much higher. We note that in our model, the mean survival rate approaches 1, though there is a large amount of variability around this mean. This high survival rate and large uncertainty is likely an artifact due to the lack of good data for older fish.

For trout cod, a linear relationship between age and survival provided a better fit than a polynomial relationship, suggesting that incidental mortality due to fishing bycatch does not markedly influence survivorship as we expected. We also note that there is a great degree of variability around the survival rates for older trout cod. This variability is likely due to a paucity of data for older fish, similar to what was found in our simulation results. When a linear or polynomial model is fitted through the age‐specific survival rates, the shape of the curve is dominated by data from younger age classes. Thus where more data are available in older ages, separate models for individual ages may be desirable.

In using otoliths along with length‐increment data, we found that using known‐age data provide a more realistic estimate of the variability in the length‐at‐age relationship. The narrow width of the credible intervals in the CMR‐only model is due to the larger numbers of individuals contributing to the generation of the random beta‐distributed $K_{i}$ parameters. For most fish, there are too few capture occasions to provide an estimate of $K_{i}$, meaning that the growth parameter for these individuals is drawn toward the mean. With known‐age data, the hyperpriors on the distribution for the $K_{i}$'s are broader, better reflecting natural variability in individual growth rates. We therefore suggest that known‐age data be incorporated where possible in the analysis. This may be accomplished using marking of juveniles or through other aging methods, either of which can be accommodated in our state‐space formulation.

Moreover, our use of a state‐space parameterization facilitates integration of other recapture or recovery data sources and makes our model easily applicable to other situations. For instance, multiple sources of capture information or a robust design analysis could be incorporated in the same framework Schwarz and Stobo (1997). We have assumed a closed population, but alternate parameterizations allowing temporary migration are similarly possible given suitable data. Another advantage of our modeling approach is that it yields age‐dependent capture parameters. These detection rates will be useful for estimating the true age structure of the population as a derived parameter, at least in closed populations, and where individual heterogeneity in capture rates is sufficiently described by age (Huggins, 1989; Link, 2003)

This flexibility of our approach produces benefits in terms of accounting for potential sources of bias such as individual heterogeneity in growth or capture rates. Bias related to uneven sampling of the population can be accounted for using hierarchical approaches such as random effects. We also suggest that accounting for age‐related differences in capture rates will deal with one highly common form of sampling bias. Heterogeneity in growth rates has also been flagged as a potential issue in growth models (Troynikov, 1998; Tyler & Rose, 1994). In our case, additional data on individual ages from otoliths clearly provide a benefit in terms of defining this uncertainty. In the case of both Murray cod and trout cod, inclusion of known‐age data leads to greater uncertainty in the estimated ages of individuals, suggesting that the interval‐only model we used may underestimate the level of variability in growth rates. We note that different parameterizations of the growth curve (including modeling of yearly or environmental effects) could place greater or lesser importance on individual differences.

In summary, linking estimates of survival rates to age has the advantage of implicitly relating survival to a measure of longevity. While the relationship between survival and age is essentially the same as that for length and age, there are many cases in which age rather than size is an important determinant of survivorship. Bringing the aspect of growth into a state‐space model provides an avenue for developing further models that recognize important ecological aspects of species’ life cycles.

## CONFLICT OF INTEREST

The authors acknowledge no potential conflict of interests.

## AUTHOR CONTRIBUTION

TB led the analysis and writing. JL led fieldwork and assisted with writing. CT assisted with growth calculations, writing of results, and design of fieldwork. ZT assisted with writing results and execution of fieldwork. SW and MM advised on statistical methods and writing.

## DATA ACCESSIBILITY

Data used in this study have been deposited into the Victoria Basin Atlas, a government‐owned database of publicly funded ecological data in Australia. https://www.environment.vic.gov.au/biodiversity/victorian-biodiversity-atlas/vba-go.

## References

[ece34633-bib-0001] Allen, M. S. , Brown, P. , Douglas, J. , Fulton, W. , & Catalano, M. (2009). An assessment of recreational fishery harvest policies for Murray cod in southeast Australia. Fisheries Research, 95, 260–267. 10.1016/j.fishres.2008.09.028

[ece34633-bib-0002] Anderson, J. R. , Morison, A. K. , & Ray, D. J. (1992). Age and growth of Murray Cod, *Maccullochella‐peelii* (Perciforms, percichthyidae), in the lower Murray‐Darling basin, Australia, from thin‐sectioned otoliths. Australian Journal of Marine and Freshwater Research, 43, 983–1013. 10.1071/MF9920983

[ece34633-bib-0003] Begg, G. A. , Hare, J. A. , & Sheehan, D. D. (1999). The role of life history parameters as indicators of stock structure. Fisheries Research, 43, 141–163. 10.1016/S0165-7836(99)00071-5

[ece34633-bib-0004] Berkeley, S. A. , Hixon, M. A. , Larson, R. J. , & Love, M. S. (2004). Fisheries sustainability via protection of age structure and spatial distribution of fish populations. Fisheries, 29, 23–32. 10.1577/1548-8446(2004)29%5b23:FSVPOA%5d2.0.CO;2

[ece34633-bib-0005] Beverton, R.J. (1954) Notes on the use of theoretical models in the study of the dynamics of exploited fish populations. 2 (pp. 159). Beaufort, NC: US Fishery Laboratory, Miscellaneous Contribution.

[ece34633-bib-0006] Beverton, R. J. H. , & Holt, S. J. (1957). On the dynamics of exploited fish populations. In, Fishery Investigations (Ser. II, Vol. XIX). London: Her Majesty's Stationery Office.

[ece34633-bib-0007] Bonner, S. J. , & Schwarz, C. J. (2006). An extension of the Cormack–Jolly–Seber model for continuous covariates with application to *Microtus pennsylvanicus* . Biometrics, 62, 142–149. 10.1111/j.1541-0420.2005.00399.x 16542240

[ece34633-bib-0008] Bouwhuis, S. , Choquet, R. , Sheldon, B. C. , & Verhulst, S. (2012). The forms and fitness cost of senescence: Age‐specific recapture, survival, reproduction, and reproductive value in a wild bird population. American Naturalist, 179, E15–E27. 10.1086/663194 22173469

[ece34633-bib-0009] Buckland, S. , Newman, K. , Thomas, L. , & Koesters, N. (2004). State‐space models for the dynamics of wild animal populations. Ecological Modelling, 171, 157–175. 10.1016/j.ecolmodel.2003.08.002

[ece34633-bib-0010] Chaozhi, Z. , Ovaskainen, O. , Saastamoinen, M. , & Hanski, I. (2007). Age‐dependent survival analyzed with Bayesian models of mark‐recapture data. Ecology, 88, 1970–1976. 17824428 10.1890/06-1246.1

[ece34633-bib-0011] Chilvers, B. L. , & Mackenzie, D. I. (2010). Age‐ and sex‐specific survival estimates incorporating tag loss for New Zealand sea lions, phocarctos hookeri. Journal of Mammalogy, 91, 758–767. 10.1644/09-MAMM-A-285.1

[ece34633-bib-0012] Colchero, F. , Jones, O. , & Rebke, M. (2015). Basta: An r package for Bayesian estimation of age‐specific survival from incomplete mark‐recapture/recovery data with covariates. Methods in Ecology and Evolution, 3, 466–470.

[ece34633-bib-0013] Eaton, M. , & Link, W. (2012). Estimating age from recapture data: Integrating incremental growth measures with ancillary data to infer age‐at‐length. Ecological Applications, 21, 2487–2497. 10.1890/10-0626.1 22073638

[ece34633-bib-0014] Ebner, B. (2006). Murray cod an apex predator in the Murray River, Australia. Ecology of Freshwater Fish, 15, 510–520. 10.1111/j.1600-0633.2006.00191.x

[ece34633-bib-0015] Fournier, D. , Hampton, J. , & Sibert, J. R. (1998). MULTIFAN‐CL: A length‐based, age‐structured model for fisheries stock assessment, with application to South Pacific albacore, *Thunnus alalunga* . Canadian Journal of Fisheries and Aquatic Sciences, 55, 2105–2116. 10.1139/f98-100

[ece34633-bib-0016] Fournier, D. A. , Sibert, J. R. , & Terceiro, M. (1991). Analysis of length frequency samples with relative abundance data for the Gulf of Maine northern shrimp (*Pandalus borealis*) by the MULTIFAN method. Canadian Journal of Fisheries and Aquatic Sciences, 48, 591–598. 10.1139/f91-075

[ece34633-bib-0017] Francis, R. C. , & Campana, S. E. (2004). Inferring age from otolith measurements: A review and a new approach. Canadian Journal of Fisheries and Aquatic Sciences, 61, 1269–1284. 10.1139/f04-063

[ece34633-bib-0018] Huggins, R. (1989). On the statistical analysis of capture‐recapture experiments. Biometrika, 76, 133–140. 10.1093/biomet/76.1.133

[ece34633-bib-0019] King, R. (2012). A review of Bayesian state‐space modelling of capture‐recapture‐recovery data. Interface Focus, 2, 190–204. 10.1098/rsfs.2011.0078 23565333 PMC3293198

[ece34633-bib-0020] Koehn, J. D. , & Harrington, D. J. (2006). Environmental conditions and timing for the spawning of Murray cod (*Maccullochella peelii peelii*) and the endangered trout cod (*Maccullochella macquariensis*) in southeastern Australian rivers. River Research and Applications, 22, 327–342. 10.1002/(ISSN)1535-1467

[ece34633-bib-0021] Link, W. A. (2003). Nonidentifiability of population size from capture‐recapture data with heterogeneous detection probabilities. Biometrics, 59, 1123–1130. 10.1111/j.0006-341X.2003.00129.x 14969493

[ece34633-bib-0022] Lyon, J. P. , Todd, C. , Nicol, S. J. , MacDonald, A. , Stoessel, D. , Ingram, B. A. , … Bradshaw, C. J. (2012). Reintroduction success of threatened Australian trout cod (*Maccullochella macquariensis*) based on growth and reproduction. Marine and Freshwater Research, 63, 598–605. 10.1071/MF12034

[ece34633-bib-0023] Mallen‐Cooper, M. , & Stuart, I. (2003). Age, growth and non‐flood recruitment of two Potamodromous fishes in a large semi‐arid/temperate river system. River Research and Applications, 19, 697–719. 10.1002/(ISSN)1535-1467

[ece34633-bib-0024] Matechou, E. , Pledger, S. , Efford, M. , Morgan, B. J. , Thomson, D. L. , & Gimenez, O. (2013). Estimating age‐specific survival when age is unknown: Open population capture‐recapture models with age structure and heterogeneity. Methods in Ecology & Evolution, 4, 654 10.1111/2041-210X.12061

[ece34633-bib-0025] Maunder, M. , & Punt, A. (2013). A review of integrated analysis in fisheries stock assessment. Fisheries Research, 142, 61–74. 10.1016/j.fishres.2012.07.025

[ece34633-bib-0026] McCrea, R. S. , Morgan, B. J. T. , & Cole, D. J. (2013). Age‐dependent mixture models for recovery data on animals marked at unknown age. Journal of the Royal Statistical Society Series C‐Applied Statistics, 62, 101–113. 10.1111/j.1467-9876.2012.01043.x

[ece34633-bib-0027] Meixell, B. , Lindberg, M. , Conn, P. , Dau, C. , Sarvis, J. , & Sowl, K. (2013). Age‐specific survival of tundra swans on the lower Alaska Peninsula. Condor, 115, 280–289. 10.1525/cond.2013.110213

[ece34633-bib-0028] Péron, G. , Crochet, P. A. , Choquet, R. , Pradel, R. , Lebreton, J. D. , & Gimenez, O. (2010). Capture–recapture models with heterogeneity to study survival senescence in the wild. Oikos, 119, 524–532. 10.1111/j.1600-1706.2009.17882.x

[ece34633-bib-0029] Plummer, M. JAGS: A program for analysis of Bayesian graphical models using Gibbs sampling. In, *Proceedings of the 3rd international workshop on distributed statistical computing * 2003 Mar 20 (124, No. 125.10).

[ece34633-bib-0030] Pollock, K.H. (1981) Capture‐recapture models allowing for age‐dependent survival and capture rates. Biometrics, 37, 521 10.2307/2530565

[ece34633-bib-0031] R Development Core Team . (2013). R: A language and environment for statistical computing. Vienna, Austria: R Foundation for Statistical Computing.

[ece34633-bib-0032] Rowland, S. (1998). Age and growth of the Australian freshwater fish, Murray cod, *Maccullochella peeli peeli* . Linnaean Society of New South Wales, 120, 163–180.

[ece34633-bib-0033] Schmaltz, L. , Cezilly, F. , & Bechet, A. (2011). Using multistate recapture modelling to assess age‐specific bottlenecks in breeding success: A case study in the greater amingo *Phoenicopterus roseus* . Journal of Avian Biology, 42, 178–186. 10.1111/j.1600-048X.2010.05112.x

[ece34633-bib-0034] Schwarz, C. J. , & Stobo, W. T. (1997). Estimating temporary migration using the robust design. Biometrics, 53, 178–194. 10.2307/2533106

[ece34633-bib-0035] Su, Y.S. , & Yajima, M. (2011) R2jags: A Package for Running jags from R. R package version 0.02‐15.

[ece34633-bib-0036] Troynikov, V. S. (1998). Probability density functions useful for parametrization of heterogeneity in growth and allometry data. Bulletin of Mathematical Biology, 60, 1099–1122. 10.1006/bulm.1998.0058

[ece34633-bib-0037] Tyler, J. A. , & Rose, K. A. (1994). Individual variability and spatial heterogeneity in fish population models. Reviews in Fish Biology and Fisheries, 4, 91–123. 10.1007/BF00043262

[ece34633-bib-0038] Von Bertalanffy, L. (1957). Quantitative laws in metabolism and growth. Quarterly Review of Biology, 32, 217–231. 10.1086/401873 13485376

